# Improved Optical Path Structure for Symmetric Demodulation Method in EFPI Fiber Optic Acoustic Sensors Using Wavelength Division Multiplexing

**DOI:** 10.3390/s23104985

**Published:** 2023-05-22

**Authors:** Hao Chen, Chenggang Guan, Hui Lv, Can Guo, Shiyi Chai

**Affiliations:** 1 School of Science, Hubei University of Technology, Wuhan 430068, China; 2 Hubei Engineering Technology Research Center of Energy Photoelectric Device and System, Hubei University of Technology, Wuhan 430068, China

**Keywords:** EFPI fiber optic sensor, wavelength division multiplexing, three-wavelength phase demodulation

## Abstract

This paper presents a novel improvement in the optical path structure of a three-wavelength symmetric demodulation method applied to extrinsic Fabry–Perot interferometer (EFPI) fiber optic acoustic sensors. The traditional approach of using couplers to construct the phase difference in the symmetric demodulation method is replaced with a new approach that combines the symmetric demodulation algorithm with wavelength division multiplexing (WDM) technology. This improvement addresses the issue of a suboptimal coupler split ratio and phase difference, which can affect the accuracy and performance of the symmetric demodulation method. In an anechoic chamber test environment, the symmetric demodulation algorithm implemented with the WDM optical path structure achieved a signal-to-noise ratio (SNR) of 75.5 dB (1 kHz), a sensitivity of 1104.9 mV/Pa (1 kHz), and a linear fitting coefficient of 0.9946. In contrast, the symmetric demodulation algorithm implemented with the traditional coupler-based optical path structure achieved an SNR of 65.1 dB (1 kHz), a sensitivity of 891.75 mV/Pa (1 kHz), and a linear fitting coefficient of 0.9905. The test results clearly indicate that the improved optical path structure based on WDM technology outperforms the traditional coupler-based optical path structure in terms of sensitivity, SNR, and linearity.

## 1. Introduction

The external Fabry–Perot Interferometer (EFPI) fiber-optic acoustic sensor is a type of Fabry–Perot interferometer sensor that utilizes a reflective sensor structure composed of a membrane and fiber end faces, eliminating the need for signal and reference arms. In contrast, other interferometric fiber sensors such as Mach–Zehnder interferometer [1], Michelson interferometer, and Sagnac interferometer [2] require the pairing of two optical paths and belong to the category of long-arm fiber sensors. The inherent structural limitations of these interferometers make it challenging to further reduce the size of fiber-optic acoustic sensors manufactured based on them. In long-arm fiber sensors, the optical fiber serves as both a sensitive element and a light carrier, and the sensitivity of the sensor’s acoustic pressure detection is limited by the optical fiber itself.

Due to its compact optical path structure, small size, simple manufacturing process, high measurement accuracy, and high detection sensitivity [3,4], the EFPI fiber optic acoustic sensor has been widely used in various applications such as high-voltage partial discharge detection [5], pressure measurement in complex environments [6,7], temperature measurement [8], gas leakage detection [9], and ultrasonic detection [10].

The demodulation methods for the fiber optic F-P sensor are intensity demodulation and phase demodulation [11]. Intensity demodulation is basically used to fix the operation at quadrature point Q [12], which limits the dynamic range for detecting external physical quantities [13]. The demodulation operating point is sensitive to the external ambient temperature and static background pressure, resulting in Q-point drift. Alleviating drifting requires complex feedback regulation circuits, which increases the system cost [14,15]. The most common phase demodulation algorithm is a symmetric demodulation algorithm based on 3 × 3 couplers [16]. The core key of the algorithm relies on 2 × 2 and 3 × 3 couplers to construct a phase difference of 2π/3 between the three wavelengths and a splitting ratio of 1:1:1; however, this algorithm fails to achieve demodulation when the characteristics of the couplers are not ideal [17]. There have been teams that have designed new demodulation circuits for asymmetric demodulation [18], which has increased system costs. Some researchers have also used techniques such as full bias preservation structure or elliptical fitting parameter method to compensate for uneven splitting ratio and phase deviation in demodulation [19]. However, these compensation algorithms have high computational complexity and poor real-time performance, which cannot meet the requirements of practical engineering.

In this paper, we propose a three-wavelength symmetric demodulation method for optical path improvement, the biggest advantage of which is that it does not need to rely on the coupler to build the phase difference, but combines the symmetric demodulation algorithm and wavelength division multiplexing (WDM) technology to achieve the prerequisites of symmetric demodulation method by different wavelength and free spectral range (FSR) measurements. The optimized optical path structure circumvents the prerequisite of using a coupler to achieve a splitting ratio of 1:1:1. Meanwhile, for the problem of inconsistent loss of the three channels that may be caused by the use of dense wavelength division multiplexing (DWDM), the use of DWDM has been proved to have no effect on the demodulation effect through theoretical derivation and subsequent experimental data testing, and can restore the sound information to be measured. The test of audio index proves that the improved optical path structure has higher sensitivity, signal-to-noise ratio (SNR), and good linearity compared with the traditional method, which illustrates the effectiveness and good demodulation effect of the combination of WDM technology and symmetric demodulation method.

## 2. Materials and Methods

**Figure 1:**
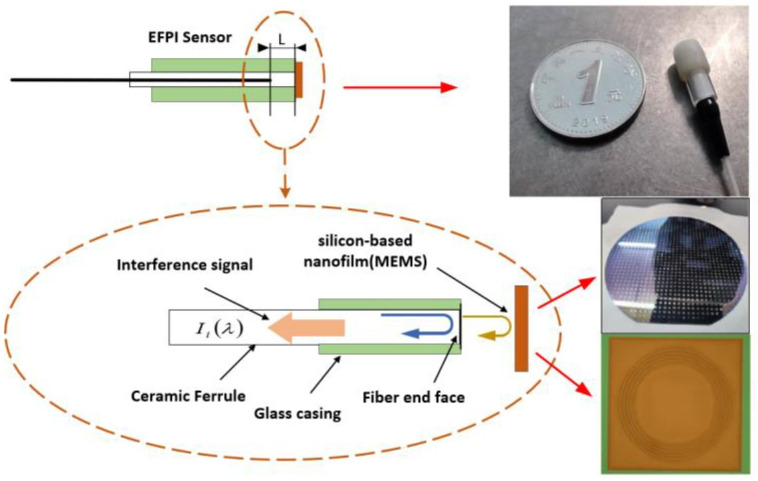
EFPI fiber optic acoustic wave sensor structure diagram and physical diagram.

The EFPI fiber optic acoustic sensor utilized in this study employs a core composed of a silicon-based nanofilm and an optical fiber endface, forming an EFPI cavity. The physical and structural characteristics of the sensor are depicted in Figure 1. The sensor structure consists of three main components: a single-mode fiber ceramic insert, a micro-electro-mechanical systems (MEMS) silicon-based nanofilm, and a glass casing. The ceramic insert measures 11 mm in length, while the glass casing has an outer diameter of 4.2 mm, an inner diameter of 2.6 mm, and a length of 10 mm, with platforms cut on both sides in the longitudinal direction. The silicon-based nanofilm, which serves as the sensitive material, is fabricated using the MEMS microfabrication processes. The core sensitive structure of the sensor is prepared through silicon etching and MEMS wafer-level bonding integration technology, as illustrated on the right side of Figure 1. A periodic annular corrugated film structure is designed to release the initial stress of the MEMS film preparation, resulting in a significant displacement of the central film under acoustic pressure, thereby greatly enhancing its acoustic detection sensitivity. Additionally, the sensor is externally protected with a sound-transparent membrane sleeve, rendering the entire sensor structure compact, small in size, passive, and metal-free. 

**Figure 2:**
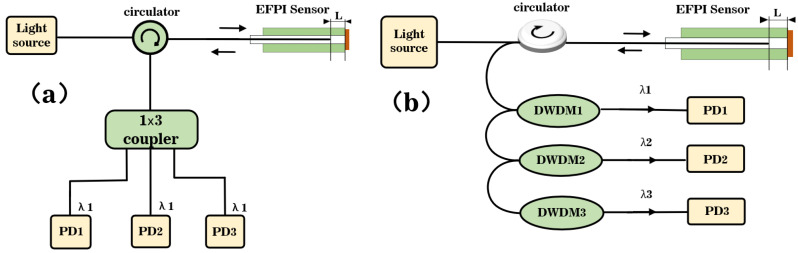
(**a**) System structure of coupler-based symmetric demodulation method. (**b**) System structure of symmetric demodulation method based on WDM technology.

The traditional three-wavelength symmetric demodulation method, based on the coupler EFPI fiber acoustic sensor system block diagram, is depicted in Figure 2a. The light emitted from the light source passes through the circulator and enters the EFPI fiber acoustic sensor for double-beam interference. The reflected light from the sensor carries the external detection signal and returns to the circulator, where it is divided into three beams by the coupler. Using the inherent characteristics of the coupler, the coupler outputs three beams of light with the same central wavelength, a light intensity ratio of 1:1:1, and a phase difference of 2π/3 between adjacent light paths. The detector then performs photodetector (PD) conversion on the three beams of interference light, and subsequently employs the principle of symmetric demodulation for phase demodulation of the three signals.

In this study, we propose an improved optical path structure based on the principle of symmetric demodulation, which eliminates the use of couplers and instead utilizes WDM technology to achieve a prerequisite phase difference of 2π/3 for the three-wavelength symmetric demodulation method. The specific approach involves observing the reflected spectrum of the EFPI fiber optic acoustic wave sensor through a spectrometer to calculate the FSR. By establishing the relationship between FSR and wavelength, the phase difference of 2π/3 between adjacent wavelengths is determined, thereby identifying the center wavelengths of the three interfering signals. By combining a broad-spectrum light source with WDM technology, three interfering signals with equal wavelength intervals are obtained. The principle of symmetric demodulation is then used to demodulate the phase change of the EFPI fiber optic acoustic wave sensor and retrieve the measured audio signal.

The block diagram of the optical path system structure for the improved three-wavelength symmetric demodulation method is illustrated in Figure 2b. A broad-spectrum light source emits light that is incident on the EFPI fiber optic acoustic wave sensor through a circulator. The interference signal reflected back by the EFPI fiber optic acoustic sensor is then directed to a DWDM via the circulator. The reflected broad-spectrum light is filtered based on wavelength, and the three interfering beams are separated accordingly. These separated beams are then converted into voltage signals through a PD and transimpedance amplification.

The silica-based nanofilm used in this study has a reflectivity of 4%, which is a weak reflective surface and constitutes a low fineness F-P cavity. According to the two-beam interference principle, three beams with central wavelengths of $\lambda_{1}$, $\lambda_{2}$, $\lambda_{3}$ and incident light intensities of $I_{1}$, $I_{2}$, and $I_{3}$ are incident on the EFPI sensor, and the light intensity expressions of the three interference signals reflected back by the sensor are:

(1) $$A_{k}=I_{k}(R_{1}+R_{2})+2I_{k}\sqrt{R_{1}R_{2}}\cos\;\left[4\;\pi \;n\;L/\lambda_{k}+\varphi \left(t\right)\right]\;\;\;\;\;k=1,\;2,\;3$$

k is the number of output light path, $A_{1}$, $A_{2}$, and $A_{3}$ are three interference signal light intensity, $\varphi \left(t\right)$ is the phase change caused by the signal to be measured, n is the air refractive index, $R_{1}$ is the fiber end-face reflectivity, $R_{2}$ is the diaphragm reflectivity. The phase difference between $A_{1}$, $A_{2}$, $A_{3}$ can be expressed as:

(2) $$\Delta \varphi_{12}=4\;\pi \;nL/\lambda_{1}-4\;\pi \;nL/\lambda_{2}\approx 2\pi \;\Delta \lambda_{12}/\mathrm{FSR}$$

(3) $$\Delta \varphi_{23}=4\;\pi \;nL/\lambda_{2}-4\;\pi \;nL/\lambda_{3}\approx 2\pi \;\Delta \lambda_{23}/\mathrm{FSR}$$

where FSR is the free spectral range of the fiber optic EFPI sensor and can be calculated from the interference spectrum reflected from the fiber optic EFPI sensor through a spectrometer. To obtain a phase difference of 2π/3 between wavelengths, the phase difference between adjacent wavelengths is constructed from the relationship between the FSR and wavelength: $\mathrm{FSR}=\frac{\lambda^{2}}{4\pi nL}$, and the relationship between the three wavelengths can be expressed as follows:

(4) $$\lambda_{2}=\lambda_{1}+\mathrm{FSR}/3$$

(5) $$\lambda_{3}=\lambda_{1}+2\mathrm{FSR}/3$$

The light intensity expressions for the three interference signals can be rewritten as follows:

(6) $$I_{k}=D_{k}+I_{k}\cos[4\;\;\pi \;nL/\lambda_{1}+\varphi (t)+(k-1)\times (2\pi /3)]\;\;\;\;\;\;\;k=1,2,3$$

$D_{k}$ is the Direct Current (DC) component of the three signals, the analog-to-digital converter (ADC) collects the voltage signal, and then removes the DC component of the voltage signal through a high-pass filter and a sliding average filter. The expressions for the conversion of three interferometric light intensities to three Alternating Current (AC) voltage signals $V_{1}$, $V_{2}$, $V_{3}$ are:

(7) $$V_{k}=\rho_{k}I_{k}\delta_{k}\cos[4\;\;\pi \;nL/\lambda_{1}+\varphi (t)+(k-1)\times (2\pi /3)]\;\;\;\;\;\;\;k=1,2,3$$

where $\rho_{1}$, $\rho_{2}$, $\rho_{3}$ are the sensitivity of the three PD; $\delta_{1}$, $\delta_{2}$, $\delta_{3}$ are the loss coefficient from the DWDM splitting to the conversion to a voltage signal in the middle of the AD acquisition. The amplitude of the three-channel voltage signal is normalized to the middle amplitude of the three channels as the normalization parameter B, then Equation (7) is rewritten as:

(8) $$C_{k}=B\cos[4\;\;\pi \;nL/\lambda_{1}+\varphi (t)+(k-1)\times (2\pi /3)]\;\;\;\;\;\;\;k=1,2,3$$

Differentiating the $C_{1}$, $C_{2}$, $C_{3}$ voltage signals to eliminate the effect of periodicity of the cos function, the following equations are obtained, respectively:

(9) $$d=-B\overset{•}{\varphi (t)}\sin[4\;\;\pi \;n\;L/\lambda_{1}+\varphi (t)]$$

(10) $$e=-B\overset{•}{\varphi (t)}\sin[4\;\;\pi \;n\;L/\lambda_{1}+\varphi (t)+2\pi /3]$$

(11) $$f=-B\overset{•}{\varphi (t)}\sin[4\;\;\pi \;n\;L/\lambda_{1}+\varphi (t)+4\pi /3]$$

For subsequent simplification, multiplying the voltage signal $C_{1}$, $C_{2}$, $C_{3}$ by the difference after differentiation of the other two optical paths gives:

(12) $$C_{1}(e-f)=\sqrt{3}B^{2}\overset{•}{\varphi (t)}\cos^{2}[4\;\;\pi \;n\;L/\lambda_{1}+\varphi (t)]$$

(13) $$C_{2}(f-d)=\sqrt{3}B^{2}\overset{•}{\varphi (t)}\cos^{2}[4\;\;\pi \;n\;L/\lambda_{1}+\varphi (t)+2\pi /3]$$

(14) $$C_{3}(d-e)=\sqrt{3}B^{2}\overset{•}{\varphi (t)}\cos^{2}[4\;\;\pi \;n\;L/\lambda_{1}+\varphi (t)+4\pi /3]$$

The equation can be simplified by applying this relationship:

(15) $$\sum_{K=1}^{3}\cos^{}\left[\varphi (t)+(k-1)\times \frac{2\pi }{3}\right]=0$$

$C_{1}\left(e-f\right),\;C_{2}\left(f-d\right),\;C_{3}\left(d-e\right)$ is added and yields N as follows:

(16) $$N=C_{1}\left(e-f\right)\;+C_{2}\left(f-d\right)\;+C_{3}\left(d-e\right)=\frac{3\sqrt{3}}{2}B^{2}\overset{•}{\varphi \left(t\right)}$$

Fluctuations in the light intensity and polarization state of the light source will lead to changes in the value of B. To avoid the effect on the demodulation effect, the voltage signal $C_{1}$, $C_{2}$, $C_{3}$ squared and obtained M:

(17) $$M=C_{1}{}^{2}+C_{2}{}^{2}+C_{3}{}^{2}=\frac{3}{2}B^{2}$$

N is divided by M and $B^{2}$ is eliminated to calculate the phase change, as follows:

(18) $$N/M=\sqrt{3}\overset{•}{\varphi \left(t\right)}$$

The phase change of the measured external signal and the calculation of the measured external signal are obtained by integrating Equation (18). The aforementioned derivation process provides a detailed account of the content of the improved algorithm. The combination of WDM technique and the principle of symmetric demodulation method is used to determine the three wavelength intervals based on the fixed phase difference of 2π/3, as deduced from the formula derivation, thus establishing the theoretical feasibility of the proposed method. In comparison to the symmetric demodulation method implemented with couplers, the improved three-wavelength symmetric demodulation method studied in this paper does not utilize couplers in the optical path, thereby circumventing the potential impact of couplers on the demodulation performance. Additionally, to ensure the effectiveness of the algorithm, the AC signal coefficients are normalized, taking into consideration the utilization of DWDM in the optical path structure, the losses introduced by the photodetector, and the errors incurred by the ADC acquisition process.

## 3. Results

### 3.1. EFPI Fiber Optic Acoustic Sensor Demodulation System Construction

**Figure 3:**
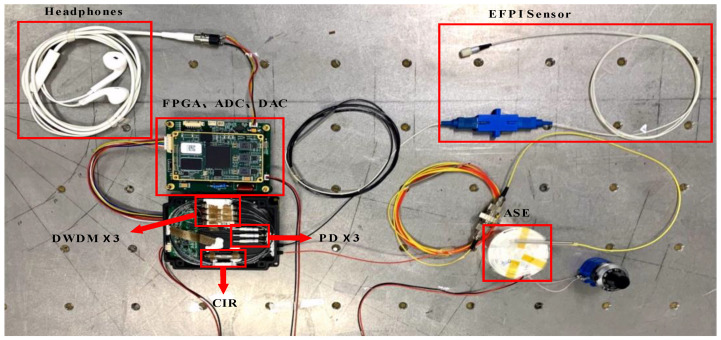
Physical diagram of EFPI fiber optic acoustic sensor demodulation system based on WDM technology.

In order to validate the effectiveness and practicality of the combined use of WDM technology and the principle of symmetric demodulation method, an optical path and circuit are designed to construct a demodulation system for fiber optic EFPI acoustic sensors based on the optical path structure depicted in Figure 2b, as illustrated in Figure 3. An amplified spontaneous emission (ASE) source with a wavelength range of 1493 to 1593 nm and a power of 50 mW is connected to a circulator (CIR-3-1550-A-025-200ZC), and the output of the circulator is connected to an EFPI fiber optic acoustic sensor. The EFPI sensor has a FSR of 12.188 nm and an interferometric contrast of 0.992. The reflected light from the EFPI sensor is directed into a three-channel DWDM system (DWDM-200G-C33-250-1-NE, DWDM-200G-C38-250-1-NE, DWDM-200G-C28-250-1- NE ) with a channel bandwidth of 200 G, which creates three interferometric signals with center wavelengths of 1546.92 nm, 1550.92 nm, and 1554.94 nm. These signals are then converted to voltage signals by three PD (PD1, PD2, and PD3) with a response of 0.85 A/W. The output voltage signals from the PD are collected by an ADC (AD7606) at a sampling frequency of 178 kHz. The real-time data captured by the ADC are transmitted through the Inter-Integrated Circuit (I2C) protocol to the field-programmable gate array (FPGA) core board (ZYNQ-7020), which demodulates the phase information of the three signals. The output of the demodulation is sent through the Serial Peripheral Interface (SPI) protocol to the digital-to-analog converter (DAC) (PCM5102), which transforms the digital audio stream into an analog audio signal.

**Figure 4:**
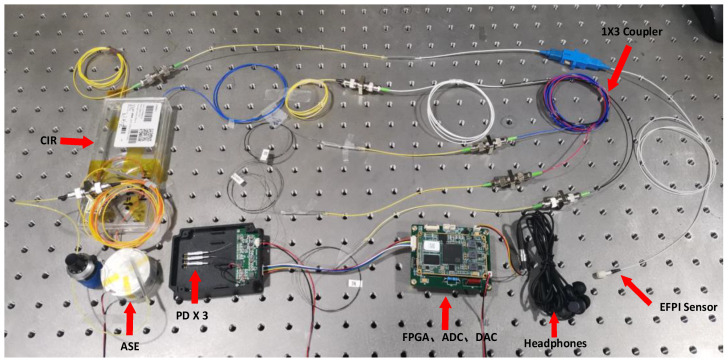
Physical diagram of the coupler-based EFPI fiber optic acoustic wave sensor demodulation system.

To compare the demodulation performance of the proposed optical path structure with the traditional coupler-based implementation of the three-wavelength symmetric demodulation method, an alternative optical path and circuit are designed to build a coupler-based EFPI fiber optic acoustic sensor demodulation system, as depicted in Figure 4, based on the optical path structure illustrated in Figure 2a.

The optical path of the coupler-based EFPI fiber optic acoustic sensor demodulation system utilizes couplers instead of three DWDMs, while the hardware circuit design remains unchanged.

### 3.2. Audio Metrics Test Environment Construction

**Figure 5:**
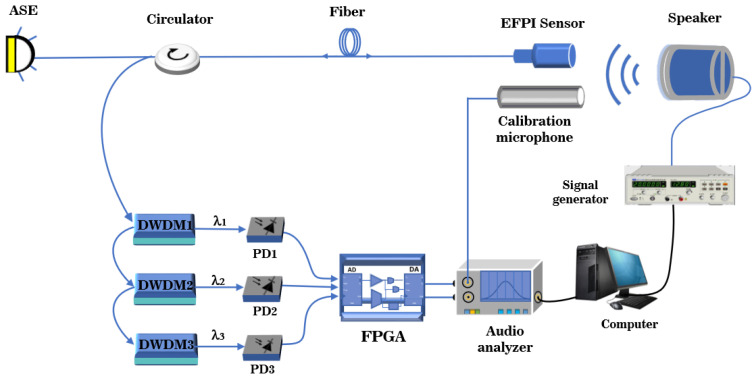
Block diagram of the audio index test system.

A demodulation audio metrics test system was built to test the actual measurement effect of the EFPI fiber optic acoustic sensor demodulation system under two different optical path structures. The block diagram of the audio metrics test system is shown in Figure 5. The system verifies the three-wavelength phase demodulation algorithm implemented under both the improved optical path structure and the coupler-based optical path structure.

**Figure 6:**
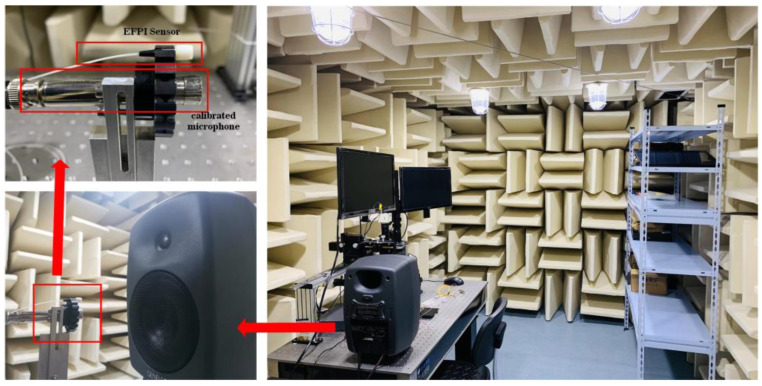
Sound-deadening indoor test platform.

The audio metrics test system utilizes a computer-controlled signal generator (ABTEC-PA01) to generate different signal frequencies and amplitudes, which are then used to drive the speaker (ABTEC-AX-MC01) to generate sound as the audio source. The analog audio signals from the calibration microphone and the fiber optic EFPI acoustic sensor demodulation systems are received by the audio analyzer (ABTEC-A2) to demodulate various audio qualities, such as signal integrity, SNR, frequency domain response, linearity, sensitivity, etc. To ensure the authenticity of the experimental data, the FP cavity probe, calibration microphone, and audio source are placed in an anechoic chamber to minimize the influence of external interference. The experimental setup in the anechoic chamber is shown in Figure 6, where the FP cavity probe and calibration microphone (HS6020) are positioned at the same distance and on the same horizontal plane as the speaker, allowing for simultaneous reception of the sinusoidal signal generated by the speaker.

### 3.3. Experiment Results 

The demodulation effect of the audio demodulation system based on wavelength division multiplexing technology was first tested. The speaker was controlled to produce a sound pressure of 0.2 Pa at an acoustic frequency of 1 kHz. The voltage signals before ADC acquisition and after demodulation were measured, where the voltage signals of the three channels before ADC acquisition contained a DC component, while the voltage signals after demodulation only contained the AC signal. The test chart is presented below.

**Figure 7:**
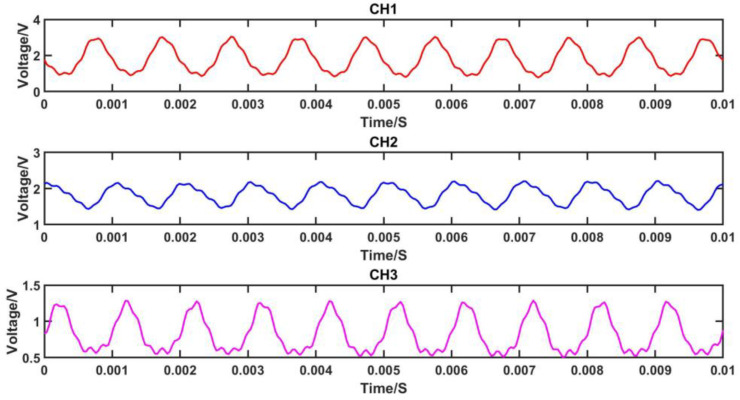
Three channels of 1 kHz signals before ADC acquisition.

From Figure 7, it can be observed that the magnitudes of the DC components of the voltage signals from the three channels before ADC acquisition are inconsistent. This inconsistency may be attributed to the varying optical intensities of the three channels due to the optical path losses introduced by the use of DWDM. However, the subsequent algorithm normalizes the signals, thereby reducing the impact on the demodulation performance.

**Figure 8:**
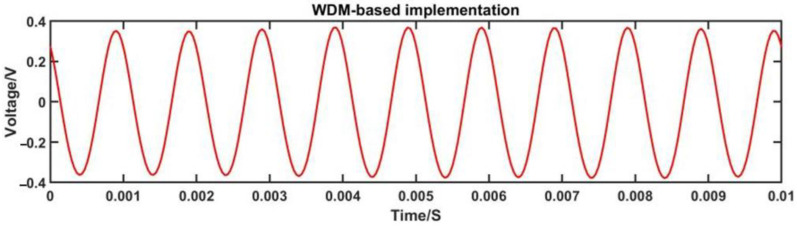
1 kHz signal after WDM-based three-wavelength demodulation.

As depicted in Figure 8, the 1 kHz signal after demodulation based on WDM exhibits a reduced amplitude compared to the voltage value before ADC acquisition, with the DC component removed. Through a comparison of the waveforms before and after demodulation in Figure 7 and Figure 8, it is evident that the signal after audio demodulation is smoother than the signal before ADC acquisition, without any distortion or abnormal jitter in the waveform. This observation suggests that the combination of wavelength division multiplexing technology and the principle of symmetric demodulation method, as implemented in the three-wavelength phase demodulation algorithm, effectively restores the detected waveform signal. The distortion in the voltage signal waveform of the three channels before ADC acquisition may be attributed to circuitry-related factors, optical path noise, or interference caused by fiber jitter.

**Figure 9:**
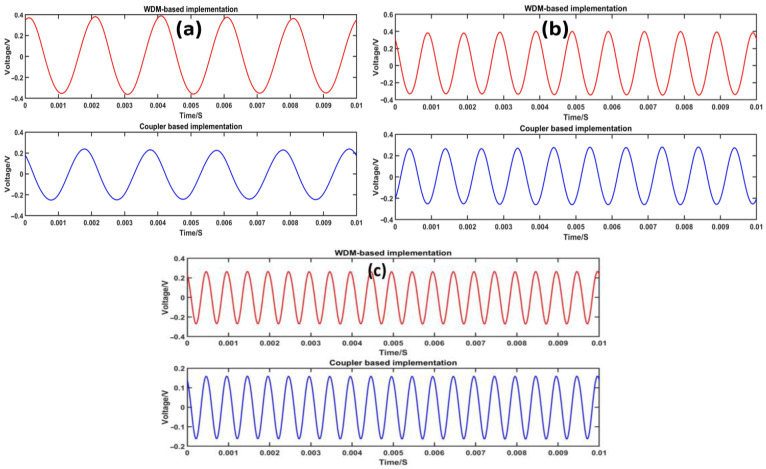
(**a**) Comparison of demodulated 0.5 kHz signal. (**b**) Comparison of demodulated 1 kHz signal. (**c**) Comparison of demodulated 2 kHz signal.

In order to investigate the demodulation capability of the three-wavelength phase demodulation method based on wavelength division multiplexing and coupler under different frequency sources, tests were conducted at varying frequencies. The signal generator was set to generate sinusoidal voltages with RMS values of 100 mV at frequencies of 500 Hz, 1 kHz, and 2 kHz, while keeping the sound pressure intensity consistent with the frequency. The time domain response comparison curves of the demodulated acoustic waves at different frequencies are presented in Figure 9.

The demodulation effects at three different frequencies, namely 0.5 kHz, 1 kHz, and 2 kHz, were compared. It was observed that, based on the same sound pressure of the source, the three-wavelength phase demodulation method using coupler and WDM technology accurately restored the measured signal at different frequencies. However, it was also noted that the amplitude of demodulation varied with the same demodulation method at different frequencies, with the mean value of three-wavelength phase demodulation using WDM slightly higher than that using coupler.

**Figure 10:**
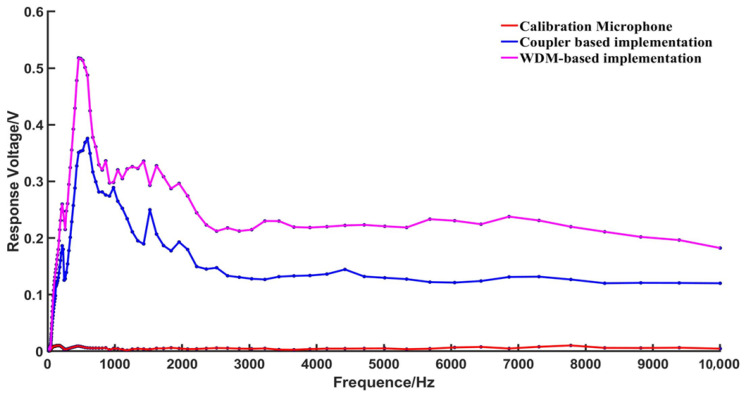
Frequency response comparison chart.

In order to investigate the specific reasons for the amplitude differences in the demodulated voltage values at different frequencies, the frequency response curve of the fiber optic EFPI acoustic sensor’s demodulation system was tested. A control signal generator generated sinusoidal signals with frequencies ranging from 20 Hz to 10 kHz, with test points selected at intervals of 50 Hz. The loudspeaker was driven with a voltage of 100 mV, and the demodulated voltage amplitude was recorded at each acoustic frequency. The frequency response of the fiber optic EFPI acoustic wave sensor’s demodulation system, employing two optical path structures, and the calibration microphone is shown in Figure 10.

The calibration microphone exhibited a comparatively flat frequency response, whereas the demodulation system of the fiber optic EFPI acoustic wave sensor with its two optical path structures displayed a significant resonance peak at a low frequency of 600 Hz. Within this resonance peak range, the demodulation system exhibited heightened sensitivity. However, the frequency response in the low-frequency region was found to be non-uniform, resulting in varying voltage amplitude of the signal response for different frequencies, even when subjected to the same sound pressure from the source.

**Figure 11:**
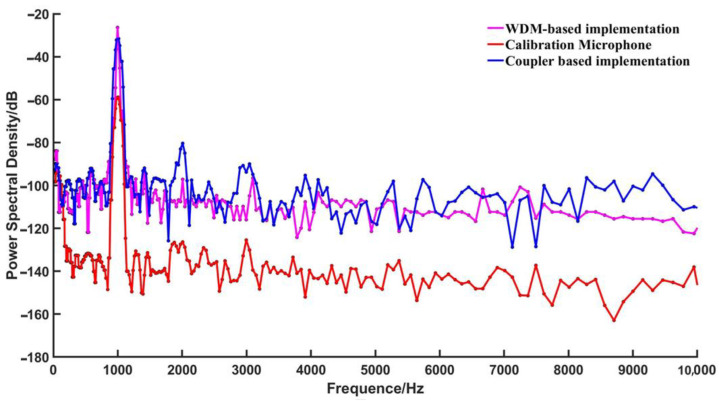
SNR comparison chart.

To investigate the impact of background noise on the demodulation performance in the demodulation system, as well as to compare the clarity and cleanliness of the demodulated sound using two different optical path structures, the power ratio between the demodulated sound signal from the fiber optic EFPI acoustic sensor and the system’s background noise was measured to calculate the SNR of the demodulation system. An external excitation signal with a frequency of 1 kHz and an amplitude of 10 mV was inputted, and the power spectrum of the system’s output for two different optical path structures is shown in Figure 11 for analysis.

The demodulation system of the WDM-based fiber optic EFPI acoustic sensor exhibited a peak power of −28.4 dB at 1 kHz, with an average bottom noise power of −103.9 dB in the frequency range of 20 Hz to 10 kHz, resulting in an SNR of 75.5 dB. On the other hand, the calibration microphone showed a peak power of −60.1 dB at 1 kHz, with an average bottom noise power of −128.3 dB in the same frequency range, yielding an SNR of 68.2 dB. In comparison, the coupler-based fiber optic EFPI acoustic sensor demodulation system demonstrated a peak power of −32.8 dB at 1 kHz, with an average noise floor power of −97.9 dB and an SNR of 65.1 dB in the frequency range of 20 Hz to 10 kHz.

**Figure 12:**
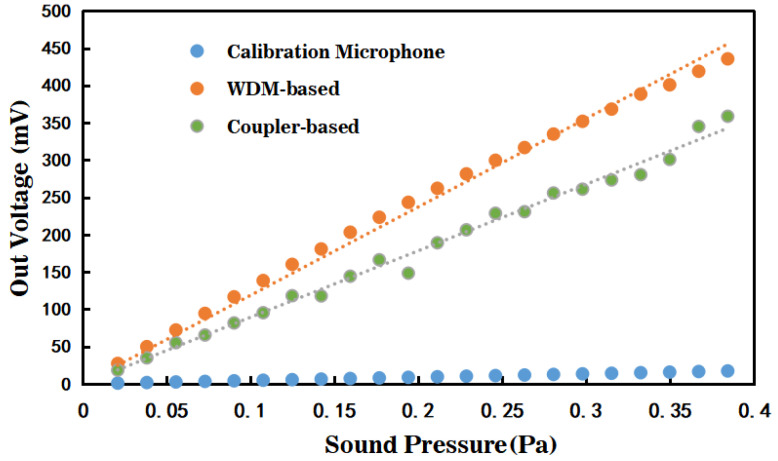
Linearity comparison chart.

To investigate the performance of the three-wavelength symmetric demodulation method in detecting external audio signals with two different optical path structures, the relationship between demodulation voltage and sound pressure intensity of the EFPI fiber optic sensor demodulation system was examined. The output voltage signals of the test fiber optic EFPI acoustic sensor demodulation system and the calibration microphone were measured at different sound pressure levels, while maintaining a constant excitation frequency of 1 kHz. The results are presented in Figure 12. Scatter plots were used to visualize the data, and a least squares linear fit was employed to test the linearity of the relationship between demodulation voltage and sound pressure intensity. The first-order linear fit expressions for the three cases are denoted by Equations (19)–(21), respectively.

The first-order linear fit based on the wavelength division multiplexing optical path structure is expressed as:

y = 1104.9x   R^2^ = 0.9946 (19)

The conventional first-order linear fit under the coupler-based optical path structure is expressed as:

y = 891.75x − 0.7213   R^2^ = 0.9905 (20)

The first-order linear fit of the calibration microphone is expressed as:

y = 45x           R^2^ = 1 (21)

In the EFPI fiber optic sensor demodulation system, the linear fitting coefficient for demodulation using WDM technique was found to be 0.9946, while the coefficient for demodulation using the coupler-based technique was 0.9905. Comparative analysis revealed that the WDM technique exhibited superior linearity and higher signal reconstruction accuracy for the three-wavelength phase demodulation. The slope of the linear fitting curve can be approximated as the demodulation sensitivity of the EFPI fiber optic sensor demodulation system. The sound pressure sensitivity of the calibration microphone in the experimental test was determined to be 45 mV/Pa, which is consistent with the technical specifications. By converting the sensitivity of the calibration microphone, the sound pressure sensitivity of the EFPI fiber optic sensor demodulation system using WDM technique was calculated to be 1104.9 mV/Pa, while using coupler-based technique was 891.75 mV/Pa. Notably, under the same source conditions, the three-wavelength phase demodulation based on WDM technology exhibited higher sensitivity and superior sound reproduction quality. The EFPI fiber optic sensor demodulation system and the calibration microphone were tested under the same experimental environment and conditions.

## 4. Discussion

**Figure 13:**
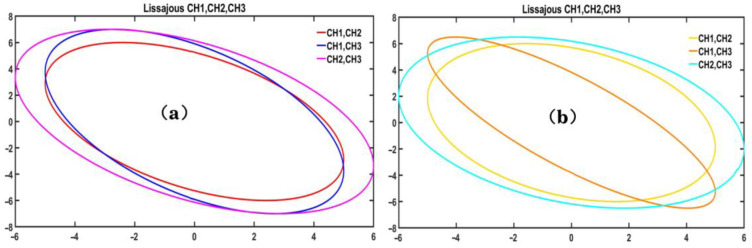
(**a**) WDM-based technology Lissajous. (**b**) Coupler-based Lissajous.

In the fiber optic EFPI acoustic wave sensor demodulation system based on WDM technology, the demodulation algorithm relies on observing the FSR of the interferometric signal to determine the phase difference between the three wavelengths. Once the complete optical path and circuit are constructed, the AC voltage signal after the PD transimpedance operational amplifier is sampled and analyzed to verify the phase relationship of the three wavelength signals in the optical path of the fiber optic EFPI acoustic wave sensor demodulation system. The frequency ratio and phase difference of the three wavelength signals were measured and plotted as depicted in Figure 13a using a Lissajous plot.

Based on the Lissajous plots, it has been observed that the phase difference between the three wavelengths, CH1 and CH2, CH1 and CH3, and CH2 and CH3, selected for the optical path of the fiber optic EFPI acoustic sensor demodulation system, is consistent with the theoretically expected value of 2π/3. This verifies the feasibility of the method proposed in this study for constructing the phase difference between adjacent wavelengths. The Lissajous plots created from CH1 and CH2, CH1 and CH3, and CH2 and CH3 exhibit overlapping ellipses of varying sizes and degrees of ellipticity, indicating that the amplitudes of CH1, CH2, and CH3 are not equal. This discrepancy may be attributed to different optical paths or circuit losses during the transmission of the three interference signals and inconsistent amplitudes of the collected AC voltage signals.

Similarly, when the coupler-based optical path structure is plotted with Lissajous diagrams, as shown in Figure 13b, it is observed that the direction of the plotted Lissajous diagram between channels CH2 and CH3 is not consistent with the other channels. Additionally, the ellipse appears to be narrower, indicating that the optical path structure implemented based on the coupler may not achieve the desired splitting ratio and phase difference.

The audio performance of the fiber optic EFPI acoustic sensor demodulation system was tested and compared with the performance of the calibration microphone in the test system under two different optical path structures. The results are presented in Table 1, and the analysis of the results is provided below. The symmetric demodulation method implemented by the improved WDM technology exhibited a significantly higher SNR of 10.4 dB compared to the traditional scheme. This improvement can be attributed to the fact that the coupler-based traditional demodulation system had a higher bottom noise of 6 dB. Furthermore, the symmetric demodulation method implemented by the improved WDM technology demonstrated the superior linearity of the demodulation effect, which closely matched the linearity of the calibration microphone. The sensitivity of the symmetric demodulation method reached 24.5 times that of the calibration microphone, indicating its higher performance.

The frequency response curves obtained from both optical path structures exhibited similar trends, indicating that the frequency response is independent of the optical path structure. However, there were differences in response amplitudes, suggesting that the optical path structure can affect the sensitivity of the audio demodulation system. The non-uniform frequency response in the demodulation system of the fiber optic EFPI acoustic sensor is likely related to the natural resonant frequency of the MEMS silicon-based nano-film, which is influenced by factors such as film material, film thickness, and area. Therefore, optimizing the acoustic structure of the MEMS film and sensor may be necessary to improve the flatness of the frequency response curve.

The higher sensitivity observed in the symmetric demodulation method implemented by the improved WDM technology may be attributed to the light intensity of each optical path. The wavelength division multiplexing technique selects three central wavelengths to divide different optical signals without affecting the optical power, whereas the coupler-based optical path structure divides the light of the same central wavelength into three paths, resulting in a division of optical power into three parts.

However, it should be noted that there are some limitations in the current research. Specifically, the bottom noise of the demodulation system for both optical path structures is higher than that of the calibration microphone. In particular, the bottom noise of the symmetric demodulation system implemented by the improved WDM technique is 24.4 dB higher than that of the calibration microphone. This suggests the possible presence of circuit noise and optical path noise in the demodulation system. Therefore, further efforts are needed to reduce the bottom noise of the system, which could involve optimizing circuit design and selecting appropriate optical–electrical devices to improve the performance of the demodulation system.

## 5. Conclusions

To provide a concise summary of this thesis work, this study proposes an optical path improvement based on a three-wavelength symmetric demodulation method for fiber optic EFPI acoustic sensors. The method utilizes the phase difference between three wavelengths to simplify the calculation of the amount of phase change within the detected audio signal. The proposed approach involves calculating the FSR by observing the interference spectrum reflected back by the EFPI sensor through a spectrometer, and then constructing the phase difference between adjacent wavelengths based on the relationship between FSR and wavelength. By determining the central wavelengths of the three interferometric signals with a phase difference of 2π/3, and obtaining the three interferometric signals with the same wavelength interval through wavelength division multiplexing, the symmetric demodulation algorithm can be used to demodulate the phase difference change of the fiber EFPI acoustic sensor and restore the measured audio signal.

This study compares the improved three-wavelength symmetric demodulation method with the traditional coupler-based symmetric demodulation method, and highlights that no coupler is used in the proposed optical path structure, thereby reducing the influence of core devices on the algorithm. In the case of the same optical power of the light source, the three-way demodulation signal of the WDM-based structure is larger than the three-way demodulation signal of the coupler-based structure, which reduces the influence of circuit and optical path noise and makes the demodulation effect better. Theoretical derivations and experimental test data indicate that the use of dense wavelength division multiplexing (DWDM) in the optical path structure does not affect the demodulation performance in terms of loss and optical device coefficients, making it conducive to the application of interferometric sensors in the field of acoustics.

Based on the theoretical framework and algorithm, the demodulation system of the fiber optic EFPI acoustic sensor, which is based on wavelength division multiplexing technology and includes a circuit design, achieves an impressive performance in terms of an SNR of 75.5 dB (1 kHz), a sensitivity of 1104.9 mV/Pa (1 kHz), and a linear fit coefficient of 0.9946 under the test environment of an anechoic chamber. Compared with the traditional coupler-based three-wavelength symmetric demodulation algorithm, the proposed method achieves a higher SNR of 10.4 dB, a lower bottom noise of the demodulation system by 6 dB, a higher demodulation sensitivity of 213.15 mV/Pa, and better demodulation linearity. Theoretical equation derivations, audio test environment, and system construction collectively confirm the effectiveness and rationality of the improved optical path structure in the three-wavelength symmetric demodulation method.

Future research can focus on reducing the system bottom noise, optimizing the acoustic structure of MEMS films and sensors, in combination with other technologies [20] to further enhance the performance and widen the applications of fiber optic EFPI acoustic sensors in the field of acoustics.

## Figures and Tables

**Table 1:** Comparison of EFPI and calibration microphone performance indicators.

	WDM-Based	Calibration Microphone	Coupler-Based
Base noise	−103.9 dB	−128.3 dB	−97.9 dB
SNR	75.5 dB	68.2 dB	65.1 dB
Linearity	0.9946	1	0.9905
Frequency response	Presence of resonance peaks	Flat	Presence of resonance peaks
Sensitivity	1104.9 mV/Pa	45 mV/Pa	891.75 mV/Pa

## Data Availability

Not applicable.
